# Solution structure of a soluble fragment derived from a membrane protein by shotgun proteolysis

**DOI:** 10.1093/protein/gzv021

**Published:** 2015-04-15

**Authors:** Mark D. Allen, Mary Christie, Peter Jones, Benjamin T. Porebski, Brendan Roome, Stefan M.V. Freund, Ashley M. Buckle, Mark Bycroft, Daniel Christ

**Affiliations:** 1 MRC Laboratory of Molecular Biology, Hills Road, Cambridge CB2 0QH, UK; 2 Department of Immunology, Garvan Institute of Medical Research,384 Victoria Street, Darlinghurst, Sydney, NSW 2010, Australia; 3 Faculty of Medicine, St Vincent's Clinical School, The University of New South Wales, Darlinghurst, Sydney, NSW 2010, Australia; 4 Department of Biochemistry and Molecular Biology, Monash University, Clayton, Victoria 3800, Australia

**Keywords:** membrane proteins, NMR spectroscopy, phage display, protein domains, proteolysis

## Abstract

We have previously reported a phage display method for the identification of protein domains on a genome-wide scale (shotgun proteolysis). Here we present the solution structure of a fragment of the *Escherichia coli* membrane protein yrfF, as identified by shotgun proteolysis, and determined by NMR spectroscopy. Despite the absence of computational predictions, the fragment formed a well-defined beta-barrel structure, distantly falling within the OB-fold classification. Our results highlight the potential of high-throughput experimental approaches for the identification of protein domains for structural studies.

## Introduction

The majority of proteins in nature are multi-domain proteins consisting of several independently folding units of structure (Liu and Rost, 2004). Domain boundaries can sometimes be predicted from sequence information alone, based on motifs or through homology with domains of known three-dimensional (3D) structure (Kelley *et al.*, 2000; Gough *et al.*, 2001; Shi *et al.*, 2001; Letunic *et al.*, 2012; Punta *et al.*, 2012). Computational approaches have thereby allowed the assignment of structural information to approximately half of all protein-coding sequences (Chothia *et al.*, 2003, Madera *et al.*, 2004). However, the domain structure of the remainder of the proteome largely remains unknown.

The limitations of computational approaches have inspired the development of experimental approaches for the identification of protein domains (Hart and Waldo, 2013). Strategies include genetic fusion with reporter proteins such as green fluorescent protein, dihydrofolate reductase or beta-lactamase (Cabantous and Waldo, 2006; Dyson *et al.*, 2008; D'Angelo *et al.*, 2011; Pedelacq *et al.*, 2011), and the use of proteolysis for high-throughput domain selection (shotgun proteolysis) (Christ and Winter, 2006). Shotgun proteolysis is based on random DNA fragmentation, followed by display of the encoded polypetides on phage, and selection for protease resistance (Christ and Winter, 2006). Based on the classic limited proteolysis method (Porter, 1959), the phage method is capable of identifying segments of structure that strongly correlate with protein domains as defined by bioinformatics predictions (Gough *et al.*, 2001; Christ and Winter, 2006). However, in addition to proteins of known structure, our previous analysis of the *Escherichia coli* proteome by shotgun proteolysis had also identified a large number of fragments of unknown structure (Christ and Winter, 2006). Many of these fragments had been derived from poorly characterized membrane or membrane-associated proteins, including a segment of the *E.coli* protein yrfF (Christ and Winter, 2006). Here we report the solution structure of this fragment as determined by NMR spectroscopy.

## Materials and methods

### Shotgun proteolysis selection

Segments of the *E.coli* W3110 genome (Bachmann, 1972) were selected by shotgun proteolysis as previously described (Christ and Winter, 2006). In brief, genomic DNA was fragmented to 300–1200 bp using a Nebulizer device (Invitrogen), repaired with Pfu polymerase and DNA adapters were ligated to the ends of the fragmentation products. Fragments were then cloned into phagemid vector pW656 and electroporated into *E.coli* TG1 (Gibson, 1984) to yield a library of 3 × 10^7^ clones. After rescue with KM13 helper phage (Kristensen and Winter, 1998), phages were incubated with 20 μg/ml of trypsin protease at 10°C for 10 min in TBS buffer supplemented with calcium (25 mM Tris–HCl, 137 mM NaCl, 1 mM CaCl_2_, pH 7.4). Protease resistant clones were captured utilizing an N-terminal barnase affinity tag, used to infect *E.coli* TG1, and analyzed by DNA sequencing.

### Expression and purification of a shotgun proteolysis fragment

For further characterization, one of the selected fragments (residues 37–154 of the *E.coli* open reading frame gi|16131275|ref|NP_417857.1| encoding the putative membrane protein yrfF, clone 14, see supplementary information of Christ and Winter (2006)) was cloned into a modified pRSETa vector (Dodd *et al.*, 2004) containing a TEV cleavage site to allow for removal of the poly-histidine tag. An additional alanine residue was incorporated at the N-terminus to facilitate TEV cleavage, resulting in a final construct incorporating residues 36–154 of yrfF.

Protein was expressed in C41 cells grown either in 2xYT medium or in K-MOPS minimal media (Neidhardt *et al.*, 1974) containing ^15^NH_4_Cl and/or [^13^C]-glucose for the production of isotopically labeled protein. Proteins were purified using Ni-NTA affinity chromatography. Following TEV cleavage and depletion by Ni-NTA affinity chromatography, a final purification step was carried out using a Superdex 75 gel-filtration column, yielding ∼30 mg of soluble protein per liter of shaking flask culture.

### NMR spectroscopy

**Fig. 1:**
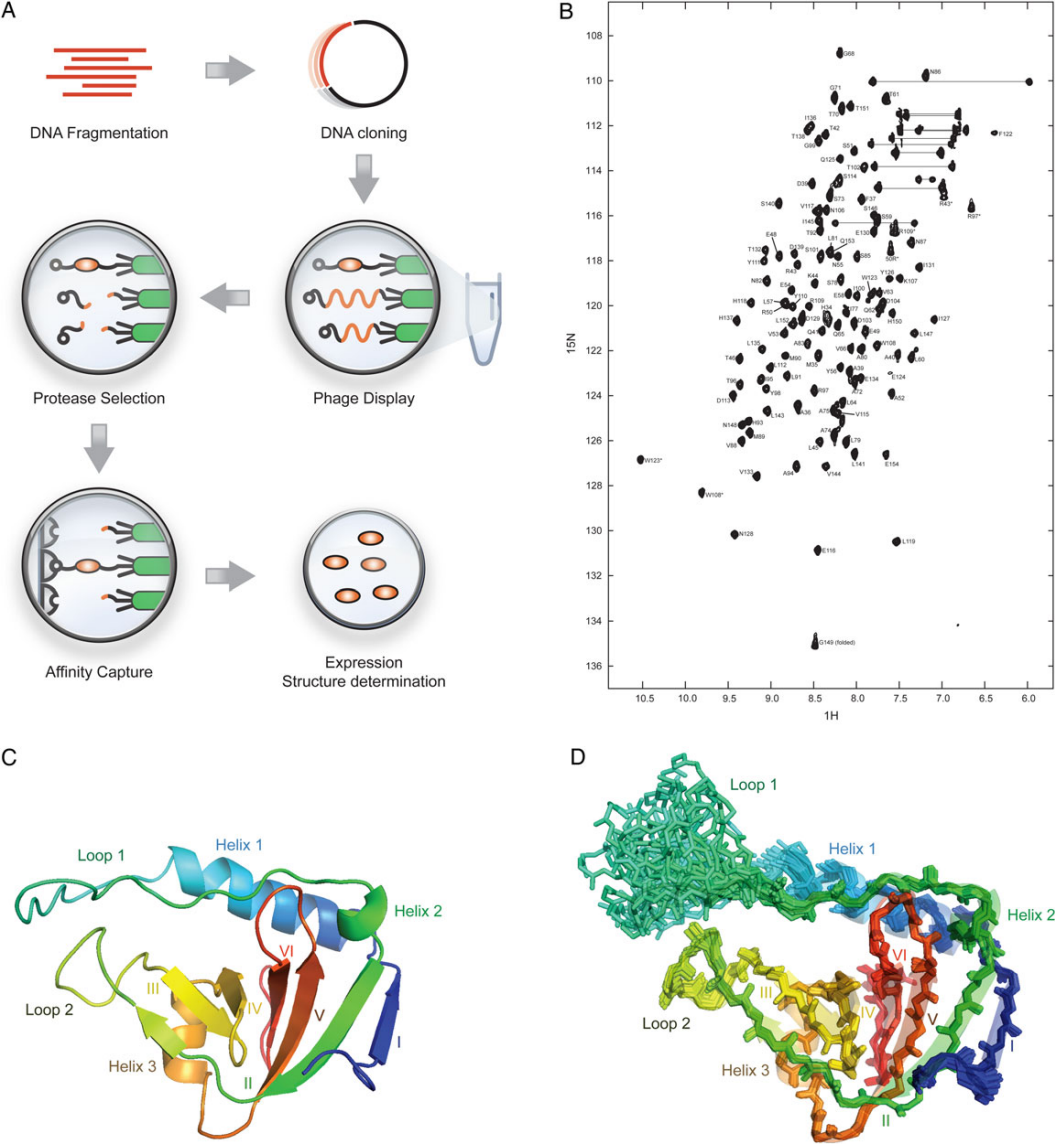
(**A**) Selection of protease resistant polypeptides by shotgun proteolysis. A library of DNA fragments were cloned into a phagemid vector, displayed on phage and protease resistant polypeptides captured using an N-terminal affinity tag. (**B**) 2D [^15^N–^1^H] HSQC spectrum of *E.coli* yrfF (residues 36–154) recorded at pH 7.0 and 293 K. The spectrum was recorded on a Bruker Avance 600 MHz spectrometer with 1024 and 512 complex points along the **t**__2__ and **t**__1__ dimensions, respectively, at a protein concentration of 1.5 m*M* in 95% H__2__O and 5% D__2__O. Peaks are labeled with single-letter amino-acid code followed by their sequence number. (**C**) NMR structure of *E.coli* yrfF (residues 36–154; PDB ID 4UZM) shown in cartoon representation. (**D**) Ensemble of backbone atoms from 20 NMR-derived structures in stick representation. Images were generated using *PyMOL*. Secondary structure elements are highlighted.

Protein samples were prepared for NMR spectroscopy experiments at a concentration of 1.5 mM in 90% H_2_O, 10% D_2_O, containing 20 mM potassium phosphate, pH 6.5, 100 mM NaCl and 5 mM β-mercaptoethanol. Spectra were acquired using Bruker DRX800 or DRX600 spectrometers equipped with pulsed ﬁeld gradient triple resonance at 25°C, and referenced relative to external sodium 2,2-dimethyl-2-silapentane-5-sulfonate for proton and carbon signals, or liquid ammonia for that of nitrogen. Assignments were obtained using standard NMR methods using ^13^C/^15^N-labeled, ^15^N-labeled, 10% ^13^C-labeled and unlabeled protein samples (Englander and Wand, 1987; Bax *et al.*, 1991). Backbone assignments were obtained using the following standard set of two-dimensional (2D) and 3D heteronuclear spectra: ^1^H–^15^N HSQC (Fig. 1), HNCACB, CBCA(CO)NH, HNCACO, HNCO, HBHA(CO)NH and ^1^H–^13^C HSQC. Additional assignments were made using 2D TOCSY and DQF-COSY spectra. A set of distance constraints were derived from 2D NOESY spectra recorded from a 1.5 mM samples with a mixing time of 100 ms. Hydrogen bond constraints were included for a number of backbone amide protons whose signals were still detected after 10 min in a 2D ^1^H–^15^N HSQC spectrum recorded in D_2_O (pH 6.5). Candidates for the acceptors were identified using the program HBPLUS for the hydrogen bond donors that were identified by the H–D exchange experiments. When two or more candidates of acceptors were found for the same donor in different structures, the most frequently occurring candidate was selected. For hydrogen bond partners, two distance constraints were used where the distance ^(D)^H–O^(A)^ corresponded to 1.5–2.5 Å and ^(D)^N–O^(A)^ to 2.5–3.5 Å. Torsional angle constraints were obtained from an analysis of C′, N, C_α_, H_α_ and C_β_ chemical shifts using the program TALOS (Cornilescu *et al.*, 1999). The stereospecific assignments of H_β_ resonances determined from DQF-COSY and HNHB spectra were confirmed by analyzing the initial ensemble of structures. Stereospecific assignments of H_γ_ and H_δ_ resonances of Val and Leu residues, respectively, were assigned using a fractionally ^13^C-labeled protein sample (Neri *et al.*, 1989). The 3D structures of the yrfF domain were calculated using the standard torsion angle dynamics-simulated annealing protocol in the program CNS 1.2 (Brunger, 2007). Structures were accepted where no distance violation was >0.25 Å and no dihedral angle violations >5° (20 accepted structures) (Table I). The backbone dynamics of the yrfF domain were investigated using steady-state {^1^H–^15^N} nuclear Overhauser enhancement (NOE) experiments (Kay *et al.*, 1989; Barbato *et al.*, 1992). Final coordinates have been deposited in the Protein Data Bank (PDB accession no. 4UZM).

**Table I.** Summary of conformational constraints and statistics

Structural constraints	
Intra-residue	992
Sequential	678
Medium-range (2 ≤ |*i* – *j*| ≤ 4)	411
Long-range (|*i* – *j*| > 4)	940
Dihedral angle constraints	36
TALOS constraints	188
Distance constraints for 44 hydrogen bonds	88
Total	3333
Statistics for accepted structures	
Statistical parameters (±SD)	
RMS deviation for distance constraints	0.0093 ± 0.0004 Å
RMS deviation for dihedral constraints	0.182 ± 0.027°
Mean CNS energy term (kcal mol^−1^ ± SD)	
*E* (overall)	200.91 ± 7.47
*E* (van der Waals)	60.95 ± 3.60
*E* (distance constraints)	20.21 ± 1.70
*E* (dihedral and TALOS constraints)	1.38 ± 0.40
RMS deviations from the ideal geometry (±SD)	
Bond lengths	0.0020 ± 0.0001 Å
Bond angles	0.422 ± 0.0070°
Improper angles	0.358 ± 0.013°
Average atomic RMSD from the mean structure (±SD)	
Residues 36–154 (N, Cα, C atoms)	0.330 ± 0.078 Å
Residues 36–154 (all heavy atoms)	0.705 ± 0.054 Å

### Sequence analysis and modeling

Iterative Hidden Markov Model (HMM) searches were performed using the JackHMMER (Finn *et al.*, 2011) web server against the UniProtKB (Magrane and Consortium, 2011) sequence database until convergence was reached. Redundancy reduction was performed with CD-HIT (Huang *et al.*, 2010). Phylogenetic trees were generated using PhyML (Guindon *et al.*, 2010). Structure predictions were performed using I-TASSER (Yang *et al.*, 2014) and EVfold (Marks *et al.*, 2011). The alignment for EVfold was built with a 99.9% redundancy-reduced alignment from the HMM search results.

## Results

### Structural features of the yrfF shotgun proteolysis fragment

**Fig. 2:**
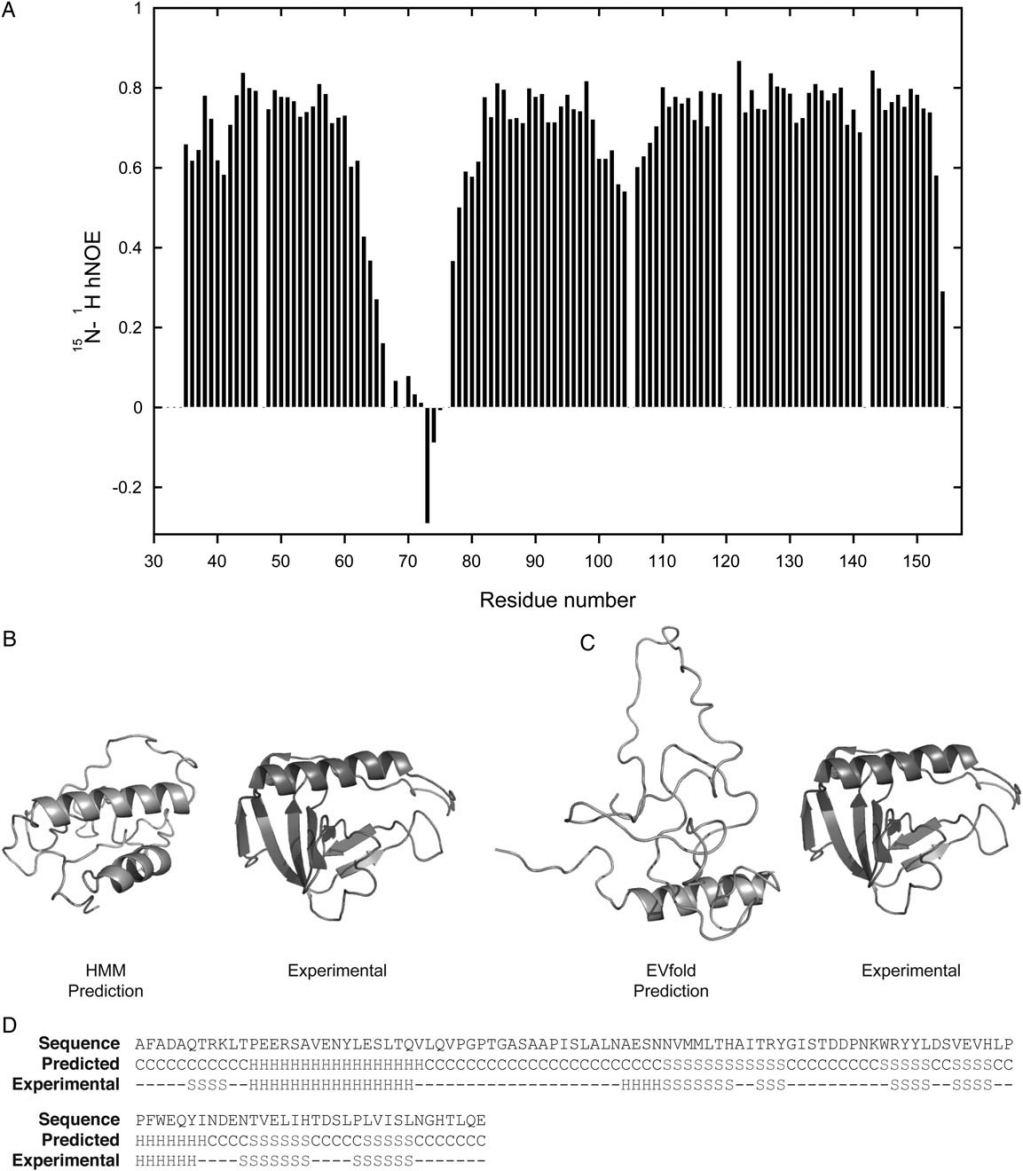
(**A**) Plot of the fractional ^1^H–^15^N heteronuclear backbone enhancement of *E.coli* yrfF (residues 36–154). Structural models of the yrfF fragment (residues 36–154) predicted from (**B**) I-TASSER and (**C**) EVfold shown in gray. For comparison, the experimental NMR structure is shown. (**D**) Secondary structure of the yrfF fragment (residues 36–154) as predicted by PsiPred and determined from the NMR ensemble. C = coil, S = beta strand, H = alpha helix.

Initial analyses revealed that the yrfF fragment identified by shotgun proteolysis (Fig. 1A) was soluble, expressed at high levels in bacteria and displayed a well-dispersed NMR ^1^H–^15^H HSQC spectrum (Fig. 1B). Further analyses revealed that a compact domain composed of a six-stranded beta-barrel (Fig. 1C). The barrel is assembled from beta-strands formed by residues 41–44 (strand I), 87–98 (strand II), 109–112 (strand III), 115–118 (strand IV), 131–137 (strand V) and 142–147 (strand VI). In addition to beta-sheet, the yrfF structure also contains three helical elements. These include a long α-helix formed by residues 47–62 (helix 1) inserted between the first and second strands of the barrel, a short helix formed by residues 83–86 (helix 2) immediately preceding strand II, and another short helix formed by residues 121–126 (helix 3) located between strands IV and V. The N- and C-termini of the domain are both highly defined, while residues within the loop region connecting helices 1 and 2 (residues 63–82) are not well defined and display an absence of long-range NOE restraints (Fig. 1D). Analysis of {^15^N–^1^H}-nuclear Overhauser enhancement values revealed that this region is indeed dynamic (Fig. 2A).

### Computational approaches fail to accurately predict the yrfF fragment structure

Although our earlier analysis of the *E.coli* genome had not revealed any structural assignments (Christ and Winter, 2006), we decided to investigate whether more recent computational methods would be capable of predicting the yrfF fragment structure. For this purpose, we utilized I-TASSER a homology approach based on HMMs (Yang *et al.*, 2014) and the evolutionary sequence variation method EVfold (Marks *et al.*, 2011). However, the two computational methods resulted in the generation of structural models, which not only differed considerably between each other, but also showed little resemblance to the structure of the yrfF fragment as determined by NMR spectroscopy (Fig. 2B and C). Indeed, both methods failed to predict any sizable part of the structure with large deviations observed (root-mean-square deviations of 10.5 Å (I-TASSER) and 14.6 Å (EVfold) over 119 C-alpha atoms). In contrast, secondary structure prediction of the yrfF fragment was broadly in line with experimental observations, although PsiPred (Jones, 1999) failed to assign several shorter elements (Fig. 2D).

### Structural analyses indicate that the yrfF fragment is a distant member of the OB-fold family

**Fig. 3:**
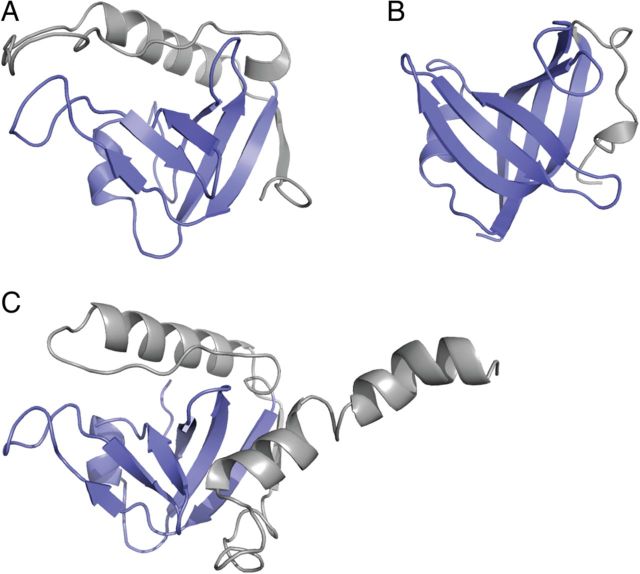
Cartoon representation of the (**A**) yrfF fragment and (**B**) *S.tokodaii* aspartyl-tRNA synthetase. OB-fold domains highlighted in blue. N-terminal additions to the OB-fold domain in gray. (**C**) Structural model of the distant yrfF homolog R4R201_PSEPH generated by I-TASSER using the NMR structure reported here as a template.

The absence of accurate predictions for the yrfF fragment suggested that it might form an ‘orphan fold’, with the structure reported here representing the sole representative in the PDB. However, it could also be envisaged that structural similarities might be simply be ‘hidden’ due to low sequence similarity and evolutionary divergence. To investigate this question, the searches were carried out using the Dali server and the solution structure of the yrfF fragment, to identify structural similarities with other proteins (Dietmann *et al.*, 2001). These analyses revealed distant, but detectable similarities to a range of OB-fold containing proteins, including the aspartyl-tRNA synthetase from *Sulfolobus tokodaii* (Fig. 3A and B).

The OB-fold represents a common structural class and is observed in proteins derived from all kingdoms of life; it consists of a five-stranded beta-barrel capped by an α-helical element (Arcus, 2002). This canonical structure can be observed in strands II–VI of the yrfF domain, which form an OB-fold with helix 3 serving as the capping helix. OB-folds can bind to a wide variety of biological molecules including proteins, nucleic acids and carbohydrates; this functional divergence can hinder their recognition based on sequence conservation alone (Arcus, 2002). Despite binding to a diverse range of molecules, ligands are generally bound at a common face of the OB-fold, which in the case of the yrfF domain corresponds to β-strands III and IV, and the loops connecting β-strands II–III, III–IV and V–VI.

Despite broadly falling with the OB-fold class, several features of the yrfF fragment display considerable variation from previously reported structures. This structural divergence is reflected by overall low Dali *Z*-scores (≤4.1). In particular, extended N-terminal structural features formed by strand I, helix 1 and the loop 1 region represent non-canonical variations of the OB-fold (Fig. 3A). This terminal extension (residues 36–86) embraces one side of the beta-barrel burying ∼1400 Å^2^ of the domain surface. The interaction shields hydrophobic residues on strands II, V and VI from solvent, and covers the end of the beta-barrel on the opposite face of the capping helix.

### Sequence analyses and structural assignments

In order to identify sequence homologs of the yrfF fragment, we performed iterative HMM searches against the UniprotKB database (Magrane and Consortium, 2011). This revealed a set of 5401 homologous sequences. After removal of highly conserved sequences (90% identity cutoff), a total of 145 non-redundant protein sequences were identified. These protein sequences are largely classified as members of the IgaA family and predominantly derived from gram-negative bacteria (Supplementary Fig. S1). They are devoid of structural annotations within the yrfF homology region, as determined by HMM searches of the PDB (Bernstein *et al.*, 1978), UniProt KB (Magrane and Consortium, 2011) and Pfam (PF07095) (Finn *et al.*, 2014) (excluding the structure reported here). In contrast, experimental determination of the yrfF fragment structure allowed for the assignment of structural information to this protein family. This is exemplified by the construction of a structural model (Fig. 3C) for the most distant identified homolog R4R201_PSEPH (Supplementary Fig. S1), an otherwise uncharacterized protein from *Pseudomonas protegens*.

## Discussion

Our results demonstrate that protein fragments identified by shotgun proteolysis are suitable for structural studies, as exemplified by the yrfF fragment solution structure reported here. The yrfF protein, from which the fragment had been derived from, is an inner membrane protein essential for the viability of *E.coli* (Yong *et al.*, 2013). The protein is a multi-span membrane protein and the identified shotgun proteolysis fragment corresponds to a putative cytoplasmic segment located between transmembrane helices 1 and 2. The yrfF homolog IgaA has been shown to control the transcription of a range of genes involved in the maintenance of cell wall integrity, cell division and motility (Garcia-Calderon *et al.*, 2009). IgaA plays an important role in the virulence of *Salmonella enterica* through attenuation of the Rcs system, although molecular details remain unknown (Garcia-Calderon, *et al.*, 2009). In *E.coli*, it has also recently been shown that RscF directly interacts with the yrfF periplasmic domain to trigger the Rcs phosphorelay in response to envelope stress (Cho *et al.*, 2014).

Although some aspects of the yrfF system have thereby been described in the literature, detailed mechanistic insights have so far remained elusive, possibly due to the absence of structural information for this protein family. The solution structure of the shotgun proteolysis fragment reported here not only provides molecular insights into the *E.coli* yrfF protein, but also allows the assignment of structural information to distant homologs observed in other species. Our findings highlight the potential of experimental approaches as an alternative and/or supplement to computational means for the identification of protein structure.

## Supplementary data

Supplementary data are available at *PEDS* online.

Supplementary Data
