# Supplementary material for: Solution structure of a soluble fragment derived from a membrane protein by shotgun proteolysis
Source: Protein Eng Des Sel. 2015 Apr 15;28(10):445–50. doi: 10.1093/protein/gzv021 (PMC4661788; doi:10.1093/protein/gzv021)
Supplement: Supplementary Data [file supp_28_10_445__index.html]

Solution structure of a soluble fragment derived from a membrane protein by shotgun proteolysis — Supplementary Data 

# Solution structure of a soluble fragment derived from a membrane protein by shotgun proteolysis

## Supplementary Data

Supplementary Data

**Files in this Data Supplement:**

- Supplementary Data - Pdf file
