# Supplementary figures and images for: Solution structure of a soluble fragment derived from a membrane protein by shotgun proteolysis
Source: Protein Eng Des Sel. 2015 Apr 15;28(10):445–50. doi: 10.1093/protein/gzv021 (PMC4661788; doi:10.1093/protein/gzv021)

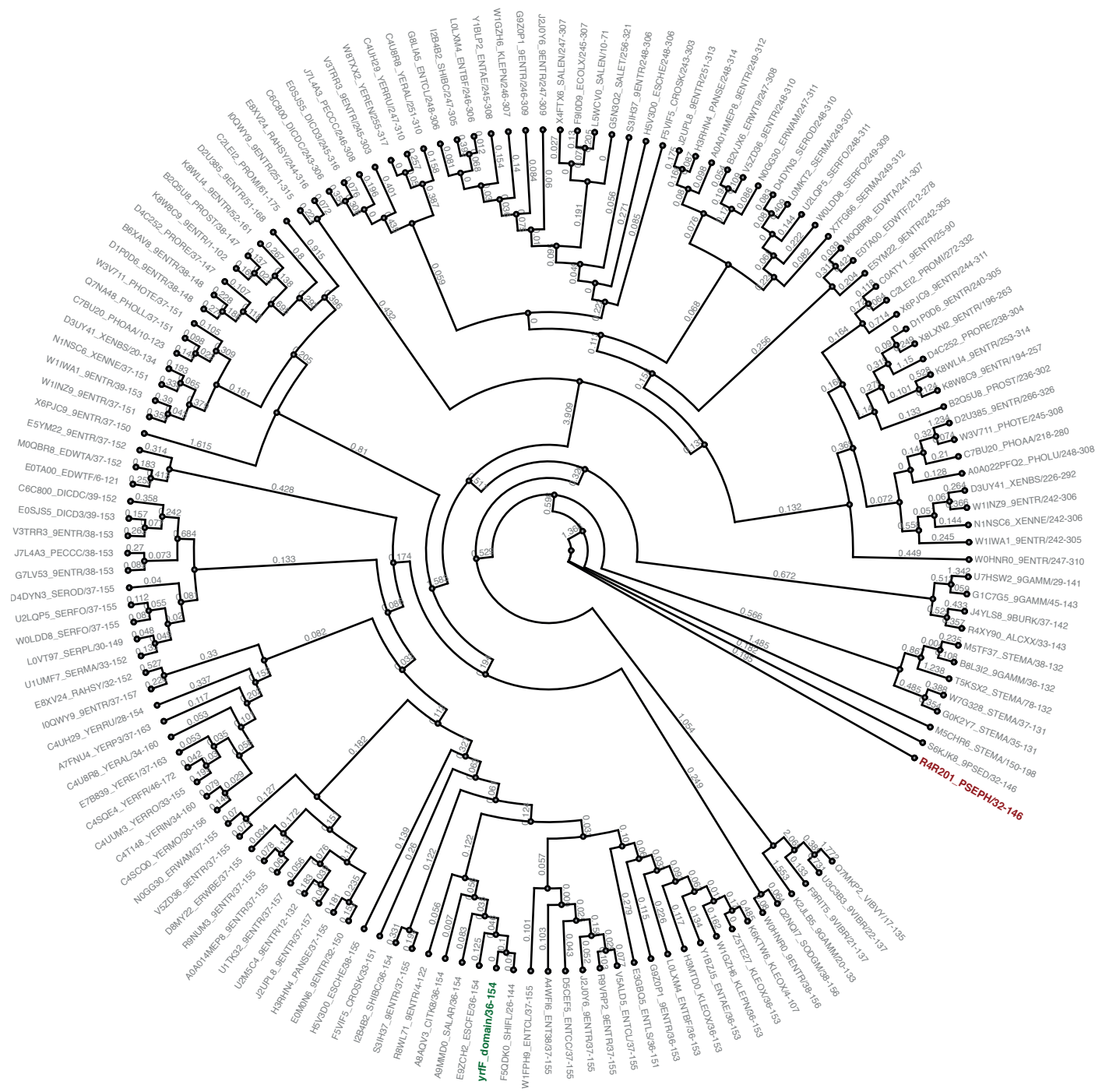

Supplement: Supplementary Data [file supp_gzv021_gzv021supp.pdf]
